# Lipoprotein A, combined with alanine aminotransferase and aspartate aminotransferase, contributes to predicting the occurrence of NASH: a cross-sectional study

**DOI:** 10.1186/s12944-020-01310-x

**Published:** 2020-06-11

**Authors:** Yu Zhang, He He, Yu-Ping Zeng, Li-Dan Yang, Dan Jia, Zhen-Mei An, Wei-Guo Jia

**Affiliations:** 1 Department of Endocrinology and Metabolism, West China Hospital, Sichuan University, Chengdu, 610041 Sichuan Province, People’s Republic of China; 2 Department of Laboratory Medicine, West China Hospital, Sichuan University, Chengdu, 610041 Sichuan China; 3 Outpatient department, West China Hospital, Sichuan University, Chengdu, 610041 Sichuan China; 4 Center for Geriatrics, West China Hospital, Sichuan University, Chengdu, 610031 Sichuan China

**Keywords:** Nonalcoholic fatty liver disease, Nonalcoholic steatohepatitis, Lipid, Lipoprotein A, Biomarkers, Diagnosis

## Abstract

**Background:**

Nonalcoholic steatohepatitis (NASH) progresses from simple nonalcoholic fatty liver (NAFL) and has a poor prognosis. Abnormal lipid metabolism is closely related to the occurrence and development of nonalcoholic fatty liver disease (NAFLD). This study aimed to study the relationships between serum lipid metabolites and NASH, and to improve the early diagnosis of NASH.

**Methods:**

This study included 86 NAFLD patients (23 NASH and 63 NAFL), and 81 unaffected individuals as controls from West China Hospital between October 2018 and May 2019. With lipid metabolites as the focus of the study, the differences in lipid metabolites were compared between the control group, NAFL patients, and NASH patients. Logistic regression analysis was used to examine the risk factors of NASH. Finally, receiver operating characteristic curve (ROC curve) was used to analyze the efficacy of the metabolites in NASH prediction.

**Results:**

The levels of alanine aminotransferase (ALT), aspartate aminotransferase (AST), and lipoprotein A (LPA) increased with the severity of NAFLD. In NAFLD patients, LPA (OR:1.61; 95%CI: 1.03–2.52) was a potential risk factor for NASH, and ROC analysis showed that the combination of LPA, ALT, and AST had a greater predictive efficiency for NASH.

**Conclusions:**

Abnormal apolipoprotein/lipoprotein is closely related to lipid metabolism disorder in patients with NAFLD. In NAFL, the combination of LPA, ALT, and AST contributes to predicting the occurrence of NASH. LPA may be a potential biomarker and therapeutic target for diagnosing and treating NASH.

## Introduction

Nonalcoholic fatty liver disease (NAFLD) is a clinical and pathological syndrome characterized by excessive intracellular fat deposition [1], which is closely related to heredity and environment [2]. NAFLD is an increasingly global health problem owing to lifestyle changes [3]. The prevalence of NAFLD is reported to be 20–30% in Western countries and 15–20% in Asian countries [4, 5], and NAFLD is expected to be a major predictor for liver transplantation in the future [6].

NAFLD includes two pathologically distinct conditions with different prognoses: nonalcoholic fatty liver (NAFL) and nonalcoholic steatohepatitis (NASH) [7]. NAFL refers to liver steatosis (fatty liver) alone, and NASH is defined as a more severe condition with inflammation and hepatocellular damage (steatohepatitis). NAFL rarely progresses [8], whereas NASH can lead to cirrhosis and even liver cancer [4]. This differential prognosis makes early identification of NASH and NAFL important. Abnormal lipid metabolism is the main cause of NAFLD [9, 10]. Therefore, this study compared and analyzed the blood lipid metabolites of the normal population and NAFLD patients (including NASH and NAFL patients). This study aimed to examine the predictive value of serum lipid metabolites in NASH, and to improve the early detection of NASH.

## Material and methods

### Patients

A total of 86 patients with NAFLD were recruited from West China Hospital between October 2018 and May 2019. These patients were categorized into two groups: 23 patients with NASH and 63 patients with NAFL. A total of 81 individuals were recruited from the physical examination center of West China Hospital as the control group.

NAFLD is the presence of hepatic steatosis [11, 12]. In this study, liver steatosis was diagnosed using abdominal ultrasound. The clinically experienced imaging physician was unaware of the subject’s clinical diagnosis and biochemical tests. From liver biopsy results, NASH was diagnosed according to the NAFLD activity score (NAS:0–8) given in the NASH clinical research network guidelines. NASH was defined as NAS ≥ 3 and histologically diagnosed as steatohepatitis. NAFL was defined as an NAS of less than or equal to 2 points in the absence of balloon degeneration [13]. Each participant underwent liver ultrasound, and the diagnosis of each NASH patient was confirmed by liver biopsy. For patients who had NAFLD but without indications of liver biopsy, experienced clinicians will comprehensively judge the condition of the patients based on the results of ultrasound examination, hepatic biochemical indicators, and hepatic transient elastic imaging techniques (e.g., Fibroscan), and include the patients meeting the requirements in NAFL group.

The exclusion criteria are presented in Table 1.

**Table 1:** Exclusion criteria

Exclusion criteria	1) more than 20 g/day of ethanol intake
	2) cirrhosis
	3) other liver diseases (viral hepatitis, autoimmune hepatitis, drug-induced liver injury, etc.)
	4) pancreatitis
	5) severe anemia
	6) acute and chronic kidney disease
	7) cancer
	8) pregnancy
	9) drug addiction
	10) use of the following drugs in the 12 months prior to screening: anti-diabetic drugs, anti-hypertensive drugs, anti-dyslipidemia drugs or folic acid and B vitamins

This study protocol conforms to the ethical guiding principles of The Declaration of Helsinki, obtaining the informed consent of the participants and the approval of the Biomedical Ethics Committee of West China Hospital of Sichuan University [Approval (no. 977) in 2019].

### Laboratory measurements

Research subjects were tested using dawn fasting venous blood line of alanine aminotransferase (ALT), aspartate aminotransferase (AST), fasting plasma glucose (FPG), fasting insulin (FINS), triglycerides (TG), total serum cholesterol (TC), high-density lipoprotein (HDL), low-density lipoprotein (LDL), apolipoprotein A1 (ApoA1), apolipoprotein B (ApoB), lipoprotein A (LPA) and homocysteine (Hcy). ALT, AST, HDL, and LDL, and other biochemical indicators were detected by Roche’s automatic biochemical analyzer and the corresponding kit (Roche, Mannheim, Germany). ApoA1 and ApoB were detected by immunoturbidimetry (Roche, Mannheim, Germany). LPA was detected by latex-enhanced immunoturbidimetry (Roche, Mannheim, Germany). Hcy was detected by the enzyme circulation method (Maccure, Chengdu, China). In this study, the homeostasis model insulin resistance index (HOMA-IR) was used to evaluate insulin resistance. HOMA-IR was determined as [fasting insulin (mU/L) × fasting plasma glucose (mmol/L)]/22.5. At the same time, gender, age, and other indicators were registered.

### Statistical analysis

All statistical analyses were performed using statistical package for social sciences (IBM SPSS Statistics Ver. 22.0). Descriptive statistics of continuous variables are expressed as mean ± standard deviation (SD). Kolmogorov–Smirnov tests for normality of continuous data were performed. Variables such as homocysteine that were skewed were normally distributed by natural logarithm transformation before analysis. Categorical variables are expressed as numbers or fractions and compared using the chi-square (χ2) test. The continuous variables were tested by ANOVA. With adjustment for age and gender, logistic regression analysis was used to determine whether these parameters were independent risk factors for NASH, and the OR value was expressed with a 95% confidence interval (CI). The predictive efficacy of the indicators was evaluated by the ROC curve. *P* < 0.05 was considered statistically significant.

## Results

### ALT, AST, and LPA increased with the severity of NAFLD

The baseline data of all patients are summarized in Table 2. Each group was similar in gender and age. The NAFL group had higher TG, IR, ApoB, ALT, and AST levels than the control group. Among NASH and NAFL patients, there were significant differences in ALT, AST, and interestingly, in LPA.

**Table 2:** Comparison of indexes between control group, NAFL patients, and NASH patients

Projects	Control *n* = 81	NAFL *n* = 63	NASH *n* = 23	*P*^*1*^ value	*P*^*2*^ value
Age (years)	38.94 ± 5.40	38.92 ± 9.80	40.63 ± 10.65	0.99	0.42
Gender (male/female)	53/28	42/21	16/7	0.87	0.80
High blood pressure (Yes/No)	2/79	0/63	0/23	0.50	–
Diabetes mellitus (Yes/No)	2/79	2/61	0/23	0.80	0.39
FPG (mmol/L)	4.97 ± 0.46	6.01 ± 2.44	5.96 ± 1.12	< 0.001^*^	0.91
TC (mmol/L)	4.60 ± 0.68	5.17 ± 0.93	4.83 ± 1.02	< 0.001^*^	0.11
HDL (mmol/L)	3.14 ± 14.93	1.18 ± 0.33	1.14 ± 0.35	0.32	0.99
LDL (mmol/L)	2.80 ± 0.63	2.97 ± 0.85	2.92 ± 0.76	0.20	0.79
ApoA1 (g/L)	1.52 ± 0.27	1.43 ± 0.24	1.46 ± 0.27	0.06	0.54
ApoB (g/L)	0.79 ± 0.16	0.92 ± 0.20	0.92 ± 0.22	< 0.001^*^	0.76
INS (lU/mL)	6.53 ± 2.75	12.72 ± 7.41	11.95 ± 11.20	< 0.001^*^	0.63
HOMA-IR	1.46 ± 0.69	3.94 ± 3.09	4.37 ± 1.30	< 0.001^*^	0.63
Log (LPA)	2.23 ± 1.01	2.42 ± 1.22	3.22 ± 1.44	0.38	0.04^*^
Log (TG)	0.01 ± 0.36	0.77 ± 0.65	0.72 ± 0.52	< 0.001^*^	0.75
Log (Hcy)	2.40 ± 0.21	2.67 ± 0.35	2.65 ± 0.23	< 0.001^*^	0.85
Log (ALT)	3.00 ± 0.46	3.77 ± 0.66	4.30 ± 0.50	< 0.001^*^	< 0.001^*^
Log (AST)	3.01 ± 0.28	3.37 ± 0.67	3.72 ± 0.38	< 0.001^*^	< 0.001^*^

*P*^*1*^ Control vs. NAFL; *P*^*2*^ NAFL vs. NASH.

^*^The differences between the groups were statistically significant

ALT, AST, TG, Hcy, and LPA were transformed by natural logarithm

presented as mean ± standard deviation (SD)

**Fig. 1:**
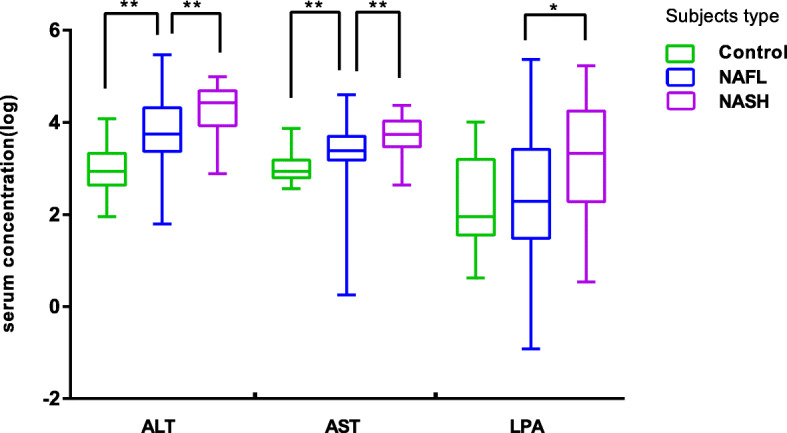
Comparison of ALT, AST, and LPA serum concentrations across groups. ** *P* < 0.01, * *P* < 0.05

According to the comparison results between groups, the indicators with obvious differences among the three groups were plotted into boxplots. As shown in Fig. 1, the levels of ALT, AST and LPA increased successively in the control group, NAFL, and NASH group.

### LPA is a potential risk factor for NASH

This study aimed to identify indicators that can help diagnose NASH from NAFLD by analyzing serum markers. Only in the comparison between NAFL and NASH, did the authors observe a significant difference in LPA levels. Therefore, logistic regression analysis was performed to verify whether LPA was a risk factor for NASH. After adjusting for gender and age, the results are shown in Table 3.

**Table 3:** Logistic analysis of NASH risk factors

Variables	^a^OR (95% CI)	*P* value
ALT	5.07 (1.72–14.99)	0.003
AST	5.68 (1.40–23.00)	0.015
LPA	1.61 (1.03–2.52)	0.039

^a^Adjusted for gender and age

### The combination of LPA, ALT, and AST was the most effective in predicting NASH

**Fig. 2:**
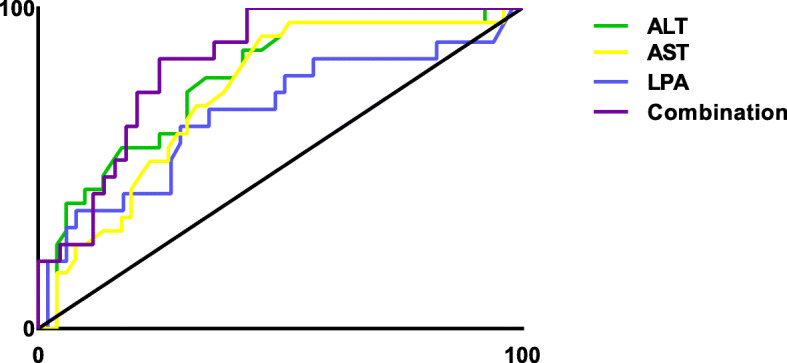
ROC of ALT, AST, and LPA and the combination indexes for NASH. The AUC of ALT was 0.77 (95%CI 0.66–0.89, *P* < 0.001), that of AST was 0.73 (95%CI 0.62 to 0.85, *P* < 0.001), that of LPA was 0.67 (95%CI 0.52 to 0.82, *P* = 0.03), and that of combination indexes was 0.83 (95%CI 0.73 to 0.93, *P* < 0.001)

Further, ROC analysis was used to evaluate the predictive efficacy of LPA on NASH, with an AUC of 0.67. Since AST and ALT levels increase with the severity of the disease, and there were significant differences between groups, on the basis of the low predictive efficacy of a single indicator, the combined diagnosis of LPA, AST, and ALT was adopted (after parameter adjustment). Including ALT, AST and LPA in the NASH risk factor regression model, the predicted probability was calculated and used as the combination index. Its predictive efficacy was the highest, with AUC of 0.83 (Fig. 2).

## Discussion

In this study, the authors found that TG and HOMA-IR levels of NAFL patients were significantly higher than those of the controls. Serum ALT, AST, and LPA levels increased with the severity of NAFLD. The authors also found that in NAFLD patients, LPA is a risk factor for NASH and the combination of LPA, ALT, and AST can predict the occurrence of NASH.

Studies have shown that insulin resistance is a key pathophysiological factor in the pathogenesis of NAFLD [14]. As a metabolic response defect [15], insulin resistance can cause disorders of lipid metabolism [16]. In the state of insulin resistance, insulin-mediated inhibition of adipocyte lipolysis and liver gluconeogenesis is weakened, resulting in increased plasma fatty acids and triglyceride synthesis in the liver [17–19]. There is an almost constant triplet reaction between Obesity-IR-NAFLD [15]. The results showed that HOMA-IR and TG levels in NAFL patients werre significantly higher than those in the control group.

This study results showed that people with NAFLD have lipid metabolism disorders, which is consistent with the results of recent studies [14, 15, 20], but its mechanism and principle have not been clearly defined. It can be seen from the clinical research results that lipid metabolism disorders are closely related to abnormal apolipoprotein/ lipoprotein.

Endogenous TG is mainly transported by ApoB [21]. ApoB levels are closely associated with fat accumulation in the liver and insulin resistance [22–25]. It is noted that there is a vicious circle between abnormal apolipoprotein, not limited to ApoB, and the accumulation of liver fat. The increase in liver fat content leads to an increase in very low-density lipoprotein (VLDL) plasma concentration, which results in higher TG, and high triglyceridemia will further promote the accumulation of liver fat [26, 27]. Conversely, increased ApoB secretion [28–30] and decreased ApoB clearance [31] due to insulin resistance can lead to elevated ApoB levels in the blood and promote the occurrence and development of NAFLD. Previous studies have indicated that plasma ApoB levels can independently predict the risk of NAFLD [32]. In this study, the authors observed higher ApoB levels in NAFL patients in comparison to the normal population.

LPA is a plasma lipoprotein composed of apolipoprotein A and cholesterol-rich LDL particles [33]. Currently, studies on LPA mainly focus on cardiovascular diseases and show that LPA is a risk factor [34–36]. A few studies have investigated the relationship between serum LPA concentration and NAFLD, but the results are contradictory [37]. By analyzing the correlation between LPA level and waist circumference, Tarantino et al. found that LPA was associated with central obesity and, more importantly, with liver fat deposition. They considered it an auxiliary risk factor for obese patients with NAFLD [38]. However, Choe et al. reported that patients with NAFLD had a lower LPA than the general population. However, after adjusting for several risk factors, the results were found only in men [39]. In this study, the authors found a positive correlation between serum LPA levels and NAFLD severity. Meanwhile, serum LPA levels were significantly different between NAFL and NASH patients, suggesting that the change in LPA concentration is specific for NASH patients. After adjusting for gender and age, regression analysis also suggested that LPA was a risk factor for NASH, which could help predict the occurrence of NASH in NAFLD patients.

Serum LPA level is the most genetically controlled of all lipoproteins, and as a quantitative genetic trait, it is widely distributed in all populations. Meanwhile, genetic variability at the LPA level is prevalent among ethnic lines. Depending on the population, approximately 30–70% of the difference in LPA concentration can be explained by LPA loci [40]. However, all subjects in this study were Han Chinese from southwest China, limiting the effect of ethnic differences on the results.

The presence of ALT, a liver enzyme, is often used to indicate the presence of liver disease. ALT is a specific marker of liver inflammation and liver cell injury. Previous studies have shown that elevated ALT levels are associated with NASH and advanced fibrosis [41]. In general, NAFLD patients with elevated ALT levels require further evaluation by a liver biopsy to determine whether they will develop NASH and advanced fibrosis [42]. However, recent observational studies have shown that patients with normal ALT may also develop NASH and progressive fibrosis, while patients with elevated ALT may have neither NASH nor progressive fibrosis [42–44]. The results suggest that the combined diagnosis of liver enzyme and LPA can improve the diagnostic efficiency of NASH, and the changes in lipid metabolism and enzymatic abnormalities can better predict the occurrence of NASH. Even if this is not enough to confirm NASH, it could provide evidence for liver biopsies.

There are several limitations to this study. First, this is a cross-sectional study that could not reflect the continuity of the same individual development. Second, the drawbacks of liver biopsy may affect the sampling results of diffuse/focal lesions. Finally, the authors cannot fully explain the clinical results observed in this study because there is no study on the mechanism of lipoprotein/apolipoprotein on NAFLD. Therefore, further studies are necessary to validate the function of LPA in NASH.

## Conclusion

Abnormal apolipoprotein/lipoprotein is closely related to lipid metabolism disorders in patients with NAFLD. LPA is a potential risk factor for NASH. In NAFLD, the combination of LPA, ALT, and AST contributes to the prediction of NASH. LPA may be a potential biomarker and therapeutic target for diagnosing and treating NASH.

## Data Availability

The datasets used and analysed during the current study are available from the corresponding author on reasonable request.

## Abbreviations

ALT: Alanine aminotransferase
AST: Aspartate aminotransferase
ApoA1: Apolipoprotein A1
ApoB: Apolipoprotein B
FINs: Fasting insulin
FPG: Fasting plasma glucose
Hcy: Homocysteine
HDL: High-density lipoprotein
HOMA-IR: Homeostatic model assessment of insulin resistance
LDL: Low-density lipoprotein
LPA: Lipoprotein A
NAFLD: Nonalcoholic fatty liver disease
NAFL: Nonalcoholic fatty liver
NASH: Nonalcoholic steatohepatitis
ROC curve: Receiver operating characteristic curve
TC: Total cholesterol
TG: Triglyceride
